# Drug Interactions Are Crucial in the Care of Patients on Opioid Substitutional Therapy—A Case Report

**DOI:** 10.3390/reports9010064

**Published:** 2026-02-14

**Authors:** Sai Keertana Devarapalli, Anna Furman-Dłubała, Agnieszka Bednarska, Justyna Dominika Kowalska

**Affiliations:** 1 English Division of the Faculty of Medicine, Medical University of Warsaw, Żwirki i Wigury 61, 02-091 Warsaw, Poland; 2 Hospital for Infectious Diseases in Warsaw, Wolska 37, 01-201 Warsaw, Polandjdkowalska@gmail.com (J.D.K.); 3 Department of Adults’ Infectious Diseases, Medical University of Warsaw, Żwirki i Wigury 61, 02-091 Warsaw, Poland; 4 Infectious Disease Observational Research Unit, Medical University of Warsaw, Żwirki i Wigury 61, 02-091 Warsaw, Poland

**Keywords:** antiretroviral therapy, buprenorphine, drug–drug interaction, opioid substitution therapy, rifampicin, tuberculosis

## Abstract

**Background and Clinical significance:** This case describes a patient with a complex medical history who develops an active *Mycobacterium tuberculosis* (MTB) infection. The complex multidrug regimen has led to significant drug–drug interactions (DDIs) and adverse effects. This case highlights an urgent need for standardized guidelines on dose adjustment and therapeutic monitoring for opioid substitution therapy (OST) and antiretroviral therapy (ART) during MTB treatment to prevent adverse health outcomes and ensure clinical success. **Case Presentation:** A 43-year-old man with medical history including human immunodeficiency virus (HIV), chronic hepatitis C virus (HCV), psychotic disorder, and opioid dependence maintained on buprenorphine (24 mg/day) presented with acute psychosis and respiratory symptoms. During hospitalization, he was diagnosed with MTB infection and was started on an empirical rifampicin-based anti-MTB regimen. His clinical course was complicated by reduced buprenorphine efficacy caused by rifampicin, which precipitated opioid withdrawal symptoms. **Conclusions:** The successful clinical stabilization with resolution of withdrawal syndrome, reduced agitation, and normalization of vital signs, including heart rate and blood pressure of this patient, was achieved through targeted management of pervasive DDIs. A strategic ART switch and careful buprenorphine dose titration during rifampicin therapy was the key factor. This case highlights that co-managing HIV, MTB, and opioid use disorder presents a significant challenge where unaddressed DDIs directly threaten treatment efficacy, a patient’s safety, and adherence, and may result in increased toxicity. The case underscores the critical need for proactive DDI assessment, interdisciplinary collaboration, and guideline development for medication optimization in people living with HIV receiving OST.

## 1. Introduction and Clinical Significance

Managing patients with opioid use disorder (OUD) is a complex process, largely due to the high burden of co-occurring medical conditions, including human immunodeficiency virus (HIV), hepatitis C virus (HCV), *Mycobacterium tuberculosis* (MTB) infection, depression, and serious bacterial infections [1]. This clinical complexity necessitates multi-drug regimens, such as opioid substitution therapy (OST), antiretroviral therapy (ART), rifampicin-based antituberculosis therapy, and antipsychotics, which increases the risk of harmful drug–drug interactions (DDIs) [2,3]. These interactions can compromise the safety and efficacy of all treatments involved and become a central determinant of clinical outcomes, potentially leading to opioid withdrawal, virological failure, and increased toxicity, thereby threatening patient stability and adherence.

Despite the known pharmacological and clinical significance of these interactions, practical guidance for managing multiple, overlapping DDIs in this population remains limited. Here, we present a case of a patient living with HIV, HCV, psychotic disorder, and on buprenorphine maintenance therapy diagnosed with MTB. This report details the cascade of DDIs encountered and the clinical management undertaken to mitigate them. The primary aim is to emphasize the importance of careful monitoring, proactive DDI assessment, dose titration, and the development of structured guidelines to optimize care for patients receiving OST within complex pharmacotherapeutic regimens.

## 2. Case Presentation

We present a 43-year-old man with a complex medical history of HIV infection, arterial hypertension, pulmonary embolism, heart failure with preserved ejection fraction, and psychotic disorders related to substance abuse. He has had a 20-year history of substance use, including tetrahydrocannabinol (THC), amphetamine, methylenedioxy-methylamphetamine (MDMA), and heroin. Since 2020, he has been enrolled in a methadone maintenance program and stabilized on buprenorphine (24 mg/day).

The patient was diagnosed with HIV in 2007 at the age of 26. Due to a history of narcotic and alcohol abuse, he was inconsistent in attending follow-up visits at the outpatient clinic, and therefore, the decision to receive ART was delayed. In 2010, at the CD4 cell count of 268 cells/mm^3^ he was prescribed ART medication consisting of emtricitabine, tenofovir, lopinavir, and ritonavir. However, the patient demonstrated poor adherence, did not take the prescribed medication, and the next HIV viral load was documented at >200 copies/mL. In 2022 he was switched to newly available integrase-based therapy, namely bictegravir, taking into account the better tolerability and DDIs profile of this therapeutic, and he continued it to the day of the latest hospitalization. He had also initiated antiviral treatment for chronic HCV with sofosbuvir and velpatasvir. The patient’s clinical course began in the third week of March, when he was initially hospitalized at the general ward of a different hospital for acute bronchitis and acute renal failure, but was discharged at his own request soon after his clinical status improved. In late March, he was readmitted due to intensifying psychotic symptoms (auditory hallucinations), most likely due to the use of illicit substances. The decision was made to start haloperidol intramuscular therapy in a dose of 50 mg per week, but due to a fear of potential QTc prolongation, buprenorphine was reduced to 4 mg/day. However, no such event was observed. Electrocardiogram (ECG) showed no signs of acute ischemia, QTc prolongation or arrhythmia, yet the patient still continued to receive a six times lower dose of buprenorphine than before admission. 

Physical examination revealed diffuse wheezing and rhonchi over the lungs, and pitting edema in the calves. Initial laboratory findings included elevated D-dimer (4868 ng/mL, reference range < 500 ng/mL), NT-proBNP (2882 pg/mL, reference range < 300 pg/mL), C-reactive protein (56.6 mg/L, reference range < 5 mg/L), IL-6 (27 pg/mL, reference range < 6.4 pg/mL), and platelets (147 G/L, reference range 125.3–396.2 G/L). A nasal swab for influenza A/B, SARS-CoV-2 and respiratory syncytial virus (RSV) was negative. Computed tomography (CT) of the chest indicated pulmonary embolism, interstitial emphysema, interstitial fibrosis, and hilar lymphadenopathy. Echocardiographic examination revealed an ejection fraction of 50%, no right ventricular (RV) overload, left ventricular (LV) hypertrophy, and signs of LV diastolic dysfunction. He received intravenous (IV) ceftriaxone, fenoterol plus ipratropium inhalations, rivaroxaban for pulmonary embolism, and antihypertensive drugs. 

After the psychotic symptoms subsided and he was stabilized, at the end of March, the patient was transferred to the Hospital of Infectious Diseases for further treatment. Upon admission, he appeared sluggish, yet periodically agitated with a blood pressure of 160/110 mmHg, heart rate > 100/min and oxygen saturation of 92%. ECG revealed no abnormalities. His buprenorphine dose was increased to 8 mg/day with significant improvement in self-reported comfort and collaboration with the clinic personnel. 

On the physical examination and CT scan of the facial skeleton without contrast, a soft oval solid-cystic lesion near the left parotid gland was found, suggestive of an enlarged lymph node exhibiting central necrosis. QuantiFERON-TB Gold Plus (QIAGEN, Hilden, Germany) test came back positive. Due to the patient’s refusal, bronchoscopy was not performed. Fine needle biopsy of the palpable lymph node identified histiocytic-macrophage elements (non-neoplastic) from which Methicillin-resistant *Staphylococcus aureus* (MRSA) was cultured, and treatment with co-trimoxazole was started. Direct microscopy from sputum and neck lymph node biopsy and polymerase chain reaction (PCR) test indicated negative *Mycobacterium tuberculosis* complex.

Based on high clinical suspicion, enlargement of the upper deep cervical lymph nodes, including jugulodigastric nodes, and a risk assumption, empirical anti-tuberculosis treatment with rifampicin, isoniazid, pyrazinamide, and ethambutol was started. The ART was switched to dolutegravir, emtricitabine, and tenofovir disoproxil due to the known rifampicin effect on bictegravir serum concentration and due to the potential risk of suboptimal treatment efficacy and an increased risk of virological failure. Within the next few days, the patient reported having strong opioid cravings, chills, sleeplessness, and general irritation. On examination, high blood pressure and heart rate, reactive pupils, and piloerection on the forearms were found. Based on the Clinical Opiate Withdrawal Scale (COWS), the patient scored 22 points, which was classified as a moderate withdrawal [4]. Due to the development of opioid withdrawal syndrome and concomitant use of buprenorphine and rifampicin, which led to accelerated metabolism of buprenorphine, the buprenorphine dose was gradually restored to 24 mg/day. The patient was closely monitored to ensure stability, and withdrawal symptoms were resolved, resulting in reduced agitation and normalization of vital signs, including heart rate and blood pressure. The patient completed hospital treatment and was discharged as planned. A timeline describing the key clinical events related to this case is summarized in Table 1.

## 3. Discussion

This case highlights the challenges in managing co-morbidities, particularly concurrent mental health issues and infections, in a patient receiving OST for substance use disorder. Three critical decision points during management profoundly influenced the treatment trajectory, with a subsequent effect on the patient’s well-being and collaboration with medical personnel (Table 2).

The first decision was to switch the patient’s ART from an integrase inhibitor, bictegravir, to a different ART regimen, with dolutegravir, emtricitabine, and tenofovir disoproxil. This decision was also made in accordance with both the European AIDS Clinical Society (EACS) and the World Health Organization (WHO) guidelines, which do not recommend the co-administration of bictegravir with rifampicin, due to potential reduced exposure of bictegravir [3,5]. This step allowed for comfortable dosing of the OST and ART.

The second critical decision was the abrupt reduction in the OST dose due to perceived drug interactions with increased cardiotoxicity. It is essential to note that, although this decision was warranted in the complex polypharmacy context, the specific concern regarding buprenorphine-associated cardiotoxicity may have been overstated. Due to its mechanism of opioid receptor interaction, buprenorphine is associated with minimal to no clinically significant QTc prolongation compared to methadone [6,7]. In this case, serial ECG monitoring confirmed the absence of significant QTc interval changes, supporting its favorable cardiac safety profile. This suggests that the perceived cardiac risk was likely due to a concurrent use of haloperidol, a known QTc-prolonging agent, rather than by buprenorphine alone [8,9]. Moreover, even though the patient was hospitalized in the psychiatric department, there was no intention to reintroduce the dose in order to provide an adequate substitution level. As a result, the patient was admitted to the infectious diseases hospital in an agitated and uneasy state and later developed an opioid withdrawal syndrome. It is crucial to acknowledge that other factors, such as the patient’s lack of adherence or the presence of concurrent illnesses could have contributed. However, increasing the dose of buprenorphine to ⅓ of the initial dose, with ECG monitoring, resulted in a significant improvement in collaboration, allowing the continuation of diagnostics. 

The final management challenge was the introduction of rifampicin treatment for MTB while sustaining ongoing ART and OST. Rifampicin, fundamental to MTB therapy and a potent inducer of the cytochrome P450 (CYP), particularly CYP3A4, reduces plasma concentration and expression of various CYP3A4 substrates [10]. This necessitated another adjustment of the ART regimen to warrant effective HIV suppression, changing from a once-daily single tablet to a twice-daily regimen of two tablets in the morning and one in the evening. Such a complex dosing schedule increases the risk of sub-optimal adherence and loss of ART effectiveness and requires close patient follow-up and informed decision-making [11,12]. 

Due to the nature of rifampicin’s broad induction effect, its interaction with OST was particularly consequential. Buprenorphine is metabolized to norbuprenorphine by CYP3A4 and CYP2C8 [13,14]. When co-administered with rifampicin, its metabolism is markedly accelerated. Studies show that this leads to a significant reduction in buprenorphine plasma concentration (70% reduction in mean Area Under the Curve (AUC)), which manifests clinically as adverse outcome, a change that clinically manifests as opioid withdrawal in 50% of patients [15,16]. Similarly, rifampicin reduces the exposure to haloperidol, another CYP3A4 substrate [10].

In contrast, the alternative rifamycin, rifabutin, demonstrates comparable antimicrobial activity to rifampicin, but acts as a less potent CYP inducer. Data shows it causes a less pronounced decrease in buprenorphine AUC (35% reduction), which correlates with an absence of clinically observed withdrawal symptoms [16]. A model-based analysis revealed that DDIs caused by low-dose rifampicin were twice as potent as those caused by rifabutin [17]. WHO guidelines also confirm the safe co-administration of rifabutin with dolutegravir-based ART [5]. Several studies and clinical guidelines, including the EACS and Centers for Disease Control and Prevention (CDC), acknowledge this interaction, often recommending rifabutin as a valid alternative [3,18]. While rifabutin is a pharmacologically superior alternative, its accessibility remains a significant barrier in Europe and worldwide [19]. Hence, the patient was not able to receive rifampicin despite the significant DDIs it causes with other medications.

Finally, the above-mentioned management strategies must be reflected in a reverse pattern while stopping rifampicin therapy. The doses of OST and haloperidol should be reduced to a minimal required dose, and the patient’s ECG should be monitored. Patient-centered care and informed collaboration are the most important factors leading to a lasting therapeutic effect.

Although DDIs involving rifampicin and OST are well recognized, practical reports showcasing the integrated management of these conditions in real clinical settings remain limited, making this case informative. Nevertheless, it is essential to consider some alternative strategies that were not feasible in this context but would be useful for future guidance. First, a proactive, pre-emptive escalation of the buprenorphine dose (from 4 mg/day to 24 mg/day) at the initiation of rifampicin therapy, rather than a reactive adjustment after onset of withdrawal, could have prevented the clinical destabilization and the reduced treatment efficacy. Second, the earlier use of rifabutin would have posed a significantly lower DDI burden, which could have prevented the opioid withdrawal symptoms and simplified management [16,17].

While this case illustrates the clinical challenges posed by DDIs between buprenorphine, ART, rifampicin, and haloperidol, it is important to note that these findings are based on a single patient report. The extrapolation of these interactions and their management to the broader population with similar co-morbidities should therefore be approached with caution. Although the pharmacokinetic basis for these interactions is well-established in the literature, robust clinical studies demonstrating effective management strategies in real-world settings remain limited.

In light of this evidence gap, the novelty of this case lies not in describing new drug interactions but in comprehensively documenting the practical clinical management and decision-making process required to manage high-risk patients with multiple overlapping DDIs in real time. While pharmacokinetic studies exist, clinical reports detailing the stepwise, interdisciplinary adjustments (e.g., ART switching, OST titration, regimen splitting) are necessary to navigate the competing risks of withdrawal, toxicity, and treatment failure. This case provides a real-world narrative that bridges theoretical interaction data with actionable clinical strategy.

The case highlights the importance of managing multiple medications in patients with comorbidities and coinfections and underscores the urgent need for more pronounced guidelines for caring for OST patients. The awareness of DDIs and their pharmacology remains a key factor to success in treating both infections and opioid dependence. Such guidelines should provide clear protocols, including strategies for dose adjustments, close clinical monitoring for withdrawal symptoms, and ideally advocating for increasing the availability of safer alternative medications like rifabutin to ensure optimal care. We hope that this study will contribute to the optimization of clinical treatment strategies and lead to improved patient outcomes.

**Table 2:** Most significant DDIs between all medications the patient received.

Drug Pair	Mechanism	Clinical Impact	Management Strategy	Outcome
Bictegravir + Rifampicin [20]	CYP3A4/UGT1A1 induction reduces bictegravir exposure.	High risk of virologic failure and emergent HIV drug resistance.	ART regimen switched to a different integrase inhibitor, Dolutegravir.	HIV viral suppression maintained and no resistance detected.
Buprenorphine + Haloperidol [21]	Potential additive QT-prolongation effects.	Increased risk of Torsades de Pointes and sudden cardiac death.	Continuous ECG monitoring and dose optimization of both drugs.	No arrhythmic events/QTc prolongation observed and therapeutic goals met.
Buprenorphine + Rifampicin [15,16]	CYP3A4 induction reduces buprenorphine exposure.	Opioid withdrawal, agitation, and risk of non-adherence.	Buprenorphine dose escalation during rifampicin therapy.	Withdrawal symptoms managed and patient stabilized.
Haloperidol + Rifampicin [10]	CYP3A4 induction reduces haloperidol plasma concentration.	Potential loss of antipsychotic effect and worsening of psychiatric symptoms.	Haloperidol dose increased during rifampicin co-administration.	Psychiatric stability maintained during treatment.
Dolutegravir + Rifampicin [20]	CYP3A4/UGT1A1 induction lowers dolutegravir plasma levels.	Increased risk of virologic failure and HIV drug resistance.	ART regimen intensified to twice-daily, multi-tablet dosing.	HIV viral load remained suppressed.

## 4. Conclusions

This case report illustrates the challenges faced and the subsequent management strategies employed in treating a patient on OST with concurrent HIV, HCV, psychiatric illness and active MTB infection. Three critical decisions shaped the clinical trajectory: appropriate switching of antiretroviral therapy to accommodate rifampicin, correcting the titration of buprenorphine dosage, and navigating rifampicin-induced interactions that triggered opioid withdrawal. This case provides a practical real-world perspective for clinicians to anticipate, identify and manage early complex DDIs in similar patient populations. It underscores the urgent need for clearer, actionable guidelines advocating proactive DDI assessment, vigilant monitoring and accurate dose strategies to optimize health outcomes in patients.

## Figures and Tables

**Table 1:** Timeline summarizing key clinical events.

Date	Intervention	Medication/DOSE Adjustment	Outcome
The third week of March	Admission to the hospital	Reduction in buprenorphine to 4 mg/day	No symptoms observed
Late March	Readmission after being discharged on demand	Uses of illicit substances outside of the hospital.	Auditory hallucinations
Late March	Admittance to the Hospital of Infectious Diseases	Reduced dose of buprenorphine for several days (4 mg/d)	The patient was agitated
The first week of April	Buprenorphine dose increased	Dose increased to 8 mg/d	Improved comfort and collaboration with personnel
The second week of April	Rifampicin therapy initiated	ARV switched to dolutegravir, emtricitabine and tenofovir disoproxil	Withdrawal symptoms started (chills, sleeplessness, general irritation, tachycardia)
Mid-April	Buprenorphine dose increased	Dose increased to 24 mg/d	Withdrawal symptoms continued
The third week of April	Discharge	None	Withdrawal symptoms resolved

## Data Availability

The original data presented in this study are available on reasonable request from the corresponding author. The data are not publicly available due to privacy concerns.
